# Review of brucellosis in Nepal

**DOI:** 10.4178/epih.e2017018

**Published:** 2017-04-24

**Authors:** Krishna Prasad Acharya, Nirajan Niroula, Krishna Kaphle

**Affiliations:** Institute of Agriculture and Animal Science, Tribhuwan University, Chitwan, Nepal

Notice of Retraction: Acharya KP et al. Review of brucellosis in Nepal. Epidemiol Health 2016;38:e2016042

The following article from Epidemiology and Health (*epi*H), “Review of brucellosis in Nepal,” published on October 1, 2016 has been retracted from publication.

The authors violated the publication ethics by their dual submission and publication to our journal and the IJVSM; International Journal of Veterinary Science and Medicine 2016;4(2):54-62: “Review of brucellosis in Nepal”. The contents were the same in papers published in both the journals. And the authors had agreed to and confirmed the “Submission Agreement”; therefore, the authors are considered to have violated *epi*H’s policy on duplicate submissions. Furthermore, it is suspected that the manuscript submitted to IJVSM is the final version that our journal had copy-edited through a professional English editing service, and also we discovered a discrepancy in the author’s name between the final version and the original submission.

Therefore, the editorial board of the *epi*H decided to retract this paper from our journal. We apologize to readers and try the best to thoroughly screen any plagiarisms and ethics violations prior to the publications of papers submitted and accepted in our journal.

